# Phytoseiid predatory mites can disperse entomopathogenic fungi to prey patches

**DOI:** 10.1038/s41598-019-55499-8

**Published:** 2019-12-19

**Authors:** Gongyu Lin, Claude Guertin, Sean-Anthony Di Paolo, Silvia Todorova, Jacques Brodeur

**Affiliations:** 1 Institut de Recherche en Biologie Végétale (IRBV), Université de Montréal, Montréal, Québec, H1X 2B2 Canada; 2 Institut national de la recherche scientifique, Centre Institut Armand-Frappier, Laval, Québec, H7V 1B7 Canada; 3 Anatis Bioprotection Inc., Saint-Jacques-de-Mineur, Québec, J0J 1Z0 Canada

**Keywords:** Agroecology, Applied microbiology

## Abstract

Recent studies have shown that predatory mites used as biocontrol agents can be loaded with entomopathogenic fungal conidia to increase infection rates in pest populations. Under laboratory conditions, we determined the capacity of two phytoseiid mites, *Amblyseius swirskii* and *Neoseiulus cucumeris* to deliver the entomopathogenic fungus *Beauveria bassiana* to their prey, *Frankliniella occidentalis*. Predatory mites were loaded with conidia and released on plants that had been previously infested with first instar prey clustered on a bean leaf. We examined each plant section to characterize the spatial distribution of each interacting organism. Our results showed that *A. swirskii* delivered high numbers of conidia to thrips infested leaves, thereby increasing the proportion of thrips that came into contact with the fungus. The effect was larger when thrips infestation occurred on young leaves than on old leaves. *Neoseiulus cucumeris* delivered less conidia to the thrips infested leaves. These patterns result from differences in foraging activity between predatory mite species. *Amblyseius swirskii* stayed longer on plants, especially within thrips colonies, and had a stronger suppressing effect on thrips than *N. cucumeris*. Our study suggests that loading certain predatory mite species with fungal conidia can increase their capacity to suppress thrips populations by combining predation and dispersing pathogens.

## Introduction

Pathogens have evolved several ways to disperse and increase the probability of encountering their host. A pathogen can be transferred directly from an infected individual to an uninfected individual, indirectly when the host encounters the free-living infectious stage of the pathogen in the environment, or via a vector^1,2^. The rate of disease transmission within a host population is strongly influenced by the spatial distribution, temporal activity pattern and foraging behaviour of interacting species (i.e. pathogens, uninfected hosts, infected hosts, vectors)^3–5^.

A growing number of studies has shown that arthropods can act as dispersal agents and transmit pathogens passively to potential hosts without becoming themselves infected^6–9^. For example, in the soil environment, collembolans can facilitate fungal dispersion by carrying conidia attached to their bodies or located in their guts^10,11^. In honeybees, phoretic Varroa mites have been identified as common vectors of viruses and fungi causing mortality and colony collapse^12,13^. Arthropod vectors therefore have the potential to shape direct and indirect interactions between a microorganism and its host and consequently influence their population dynamics, as well as the structure and stability of communities^8^. Although such interactions should be common in nature, the role of arthropod dispersal agents in pathogen epidemiology remains poorly understood.

From an applied perspective, insect pollinators and arthropod biological control agents can be used for dispersing pathogens to agricultural pests^14^, weeds^15^ and antagonists to plant diseases^16^. For example, in addition to pollinate greenhouse tomato and sweet pepper, bumble bees have the capacity to co-disseminating two fungi *Beauveria bassiana* Balsamo Vuillemin (Ascomycota: Hypocreales) and *Clonostachys rosea* (Link: Fries) Schroers, Samuels, Seifert, and Gams (Ascomycota: Hypocreales) for control of insect pests (greenhouse whitefly and tarnished plant bug) and grey mould, respectively^17^. Similarly, some species of commercially mass-produced predatory mites have shown potential for dispersing entomopathogenic fungi to insect pests. Under laboratory conditions, two phytoseiid species, *Neoseiulus cucumeris* Oudemans (Acari: Phytoseiidae) and *Amblyseius swirskii* Athias-Henriot (Acari: Phytoseiidae) facilitated the dissemination of *B. bassiana* conidia to their prey, the Asian citrus psyllid *Diaphorina citri* Kuwayama (Homoptera: Psyllidae), a major pest of citrus^18^. Such findings stimulated research on techniques to load arthropod dispersal agents with optimal doses of infective fungal conidia before releasing them in the crop where they can disseminate the pathogen to the target pests^19–21^.

While the role of host and non-host arthropods in facilitating entomopathogenic fungi dispersal in the environment has been identified^1,14^, the underlying ecological and behavioural mechanisms still need to be examined. Arthropods can mediate the rate at which a disease is horizontally transmitted to susceptible hosts through either direct physical contact (e.g. during a predation or a parasitism attempt by natural enemies) or indirectly by releasing infective propagules (fungal spores) in the habitat. In such cases, when there is a close association between the dispersal agent and the host susceptible to the pathogen, the encounter between interacting species is not a random event. For example, the capacity of a predator to disperse fungal conidia to its prey will primarily depend on how conidia are dislodged from the cuticle (either by grooming or walking) and its foraging behaviour (e.g. habitat location, area-restricted searching behaviour, numerical response) that contribute to increasing the spatial co-occurrence with the prey^22^.

This study aimed at investigating the capacity of two species of predatory mites commonly used as biological control agents in dispersing conidia of an entomopathogenic fungus to their prey. We predicted that foraging predatory mites artificially loaded with conidia will move close to their prey, thereby increasing the spatial co-occurrence between the fungus and the prey and the fungal infection rate. Under laboratory conditions, we examined the (i) spatial distribution of conidia on plant parts when unloaded from predatory mite bodies, as well as the proportion of conidia delivered to the prey oviposition leaf, (ii) predation rates and (iii) proportion of prey bearing conidia on their body. These data provide valuable insights into mechanisms involved in dispersing fungal conidia when transported by an arthropod predator that is not harmed by the fungi. They will also inform the biological control community of researchers and practitioners about the potential of predators to induce fungal epizootics in pest populations.

## Results

### Number and proportion of *B. bassiana* conidia delivered by predatory mites

**Figure 1:**
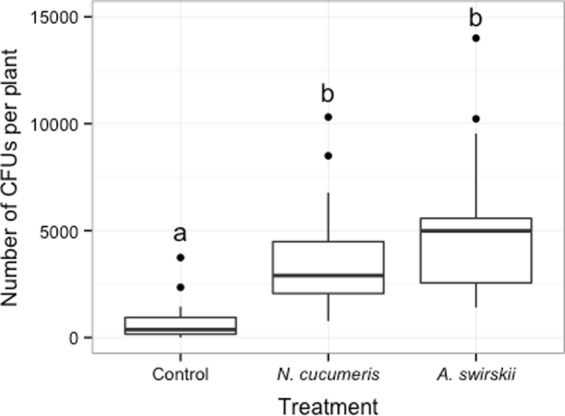
Number of *B. bassiana* colony-forming units (CFUs) recovered on a plant 48 hours after the beginning of the experiment on plants without (control) and with predatory mites, *N. cucumeris* or *A. swirskii*. Different letters indicate a significant treatment effect (p < 0.05 generalized linear model with negative binomial distribution, multiple comparisons with ‘glht’ function, Tukey method). Dots identify outliers as defined by ggplot, i.e. values exceeding 1.5 interquartile range.

The number of CFUs recovered from the entire plant significantly differed among treatments (generalized linear model with negative binomial distribution, treatment χ^2^ = 36.75, p < 0.001, Fig. 1). Both *A. swirskii* (multiple comparisons with ‘glht’ function, Tukey method, z = 6.45, p < 0.001) and *N. cucumeris* (z = 5.37, p < 0.001) contributed to increase total CFUs on plants compared to control. Both predatory mites delivered the same quantity of CFUs to the plant (z = 1.09, p = 0.519). One plant from the control treatment was excluded from the analysis because extremely high number of conidia (~19,800) landed on a single leaf; this outlier was more than three times of absolute deviation above the median^23^.

**Figure 2:**
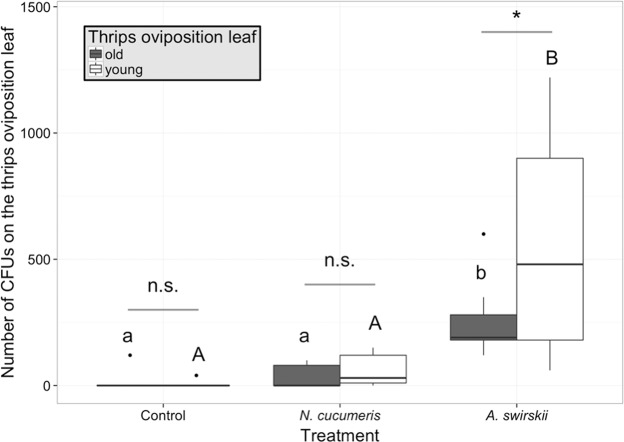
Number of *B. bassiana* colony-forming units (CFUs) recovered from the thrips oviposition leaf (young *vs*. old) 48 hours after the beginning of the experiment on plants without (control) and with predatory mites, *N. cucumeris* or *A. swirskii*. Thrips oviposition leaf refers to the leaf where thrips females were caged for 24 hours to lay eggs prior to treatments. Dots identify outliers as defined by ggplot, i.e. values exceeding 1.5 interquartile range. Different capital and lower case letters indicate significant treatment effect for young and old leaf, respectively (p < 0.05, Kruskal-Wallis test with multiple comparisons). The asterisk indicates a significant difference (0.05 < p < 0.01) between thrips oviposition leaf: n.s. = not significant (Kruskal-Wallis test within treatment ‘control’ and treatment ‘cucumeris’, generalized linear model with negative binomial distribution within treatment ‘swirskii’).

There was an interaction between the thrips oviposition leaf and treatment (generalized linear model with negative binomial distribution, interaction χ^2^ = 31.47, p < 0.001; Fig. 2). Simple effects (the effect of each independent variable within each level of the other independent variable) were examined. For *A. swirskii*, this effect was greater when thrips eggs were laid on the young leaf than on the old leaf (generalized linear model with negative binomial distribution, χ^2^ = 5.29, z = 2.32, p = 0.020). *Amblyseius swirskii* increased the number of CFUs recovered from the thrips oviposition leaf compared to control, but *N. cucumeris* did not (Kruskal-Wallis test, when thrips eggs were laid on old leaf: treatment simple effect χ^2^ = 19.81, p < 0.001; Kruskal-Wallis test, when thrips eggs were laid on young leaf: treatment simple effect χ^2^ = 18.55, p < 0.001).

**Figure 3:**
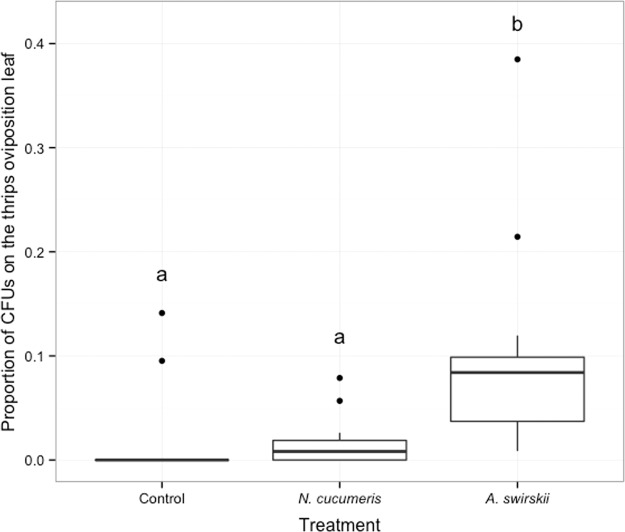
Proportion of *B. bassiana* colony-forming units (CFUs) recovered from the thrips oviposition leaf 48 hours after the beginning of the experiment on plants without (control) and with predatory mites, *N. cucumeris* or *A. swirskii*. Different letters indicate significant differences between treatments (p < 0.05, generalized linear model with normal distribution, followed by multiple comparisons with ‘glht’ function, Tukey method). Dots identify outliers as defined by ggplot, i.e. values exceeding 1.5 interquartile range.

At the beginning of the experiment, it was not possible to load the two predatory mite species with a similar number of conidia. Therefore, the proportion of *B. bassiana* delivered to the thrips oviposition leaf by the two predatory mite species was evaluated. The proportion of CFUs recovered from the thrips oviposition leaf varied among treatments (generalized linear model, χ^2^ = 23.00 p < 0.001; Fig. 3) with *A. swirskii* increasing the proportion of *B. bassiana* on the thrips oviposition leaf compared to control (generalized linear model, followed by multiple comparisons with ‘glht’ function, Tukey method, z = 4.14, p < 0.001), but not *N. cucumeris* (z = 0.06, p = 0.998). *Amblyseius swirskii* delivered a significantly higher proportion of *B. bassiana* to the thrips oviposition leaf than *N. cucumeris* (z = 4.15, p < 0.001).

### Proportion of thrips contacting *B. bassiana* delivered by predatory mites

**Figure 4:**
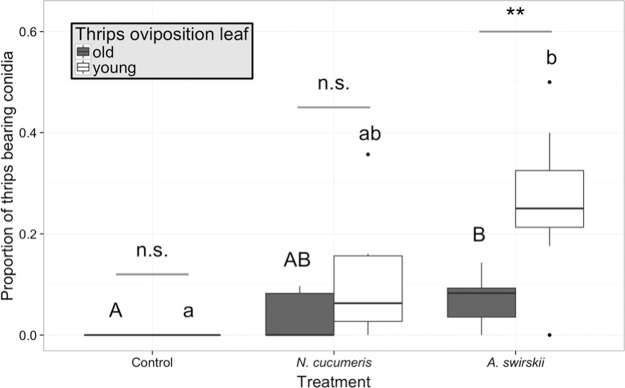
Proportion of thrips bearing *B. bassiana* 48 hours after the release of *B. bassiana* on plants without (control) and with predatory mites, *N. cucumeris* or *A. swirskii*. Thrips oviposition leaf refers to the leaf where thrips females were caged for 24 hours to lay eggs prior to treatments, old: leaf No. 5, young: leaf No. 2. Different capital letters indicate significant treatment simple effect for the young leaf (p < 0.05, generalized linear model with normal distribution, followed by multiple comparisons with ‘glht’ function, Tukey method) while different lower case letters indicate significant treatment simple effect for the old leaf (p < 0.01, generalized linear model with normal distribution, followed by multiple comparisons with ‘glht’ function, Tukey method). Differences between thrips oviposition leaves within a treatment are shown above bars: n.s. = not significant (p > 0.05), **0.001 < p < 0.01 (generalized linear model). Dots identify outliers as defined by ggplot, i.e. values exceeding 1.5 interquartile range.

The proportion of thrips coming into contact with *B. bassiana* was significantly affected by both treatment and thrips oviposition leaf (generalized linear model with normal distribution, treatment χ^2^ = 22.37, p < 0.001; oviposition leaf χ^2^ = 10.78, p = 0.001; Fig. 4), as well as by their interaction (χ^2^ = 8.00, p = 0.018). For *A. swirskii*, this effect was much greater when thrips laid eggs on the young leaf rather than the old leaf (generalized linear model with normal distribution, followed by multiple comparisons with ‘glht’ function, Tukey method, z = 3.03, p = 0.002).

### Predatory mites and thrips remaining on the plant

**Figure 5:**
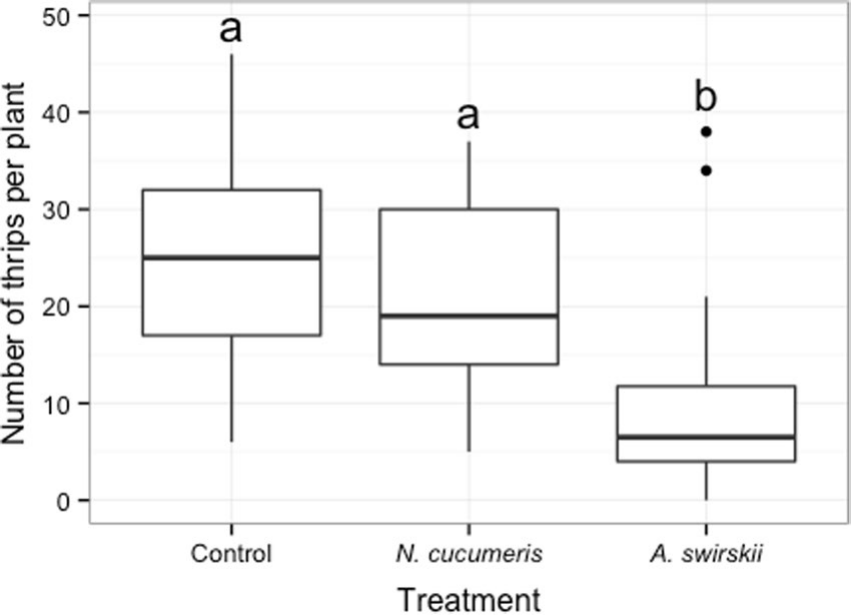
Number of thrips recovered on plant 48 hours after the beginning of the experiment on plants without (control) and with predatory mites, *N. cucumeris* or *A. swirskii*. Different letters above bars indicate significant differences between treatments (p < 0.05, generalized linear model with negative binomial distribution, followed by multiple comparisons with ‘glht’ function, Tukey method). Dots identify outliers as defined by ggplot, i.e. values exceeding 1.5 interquartile range.

Forty-eight hours following predatory mites released, higher numbers of *A. swirskii* (8.94 ± 0.88, mean ± S.E.) were recovered from the plants than *N. cucumeris* (2.61 ± 0.50 S.E.) (generalized linear model with negative binomial distribution, treatment χ^2^ = 46.82, z = 6.49, p < 0.001). The numbers of thrips recovered on plants at the end of the experiment also varied between treatments (generalized linear model with negative binomial distribution, followed by multiple comparisons with ‘glht’ function, Tukey method, χ^2^ = 15.92, p < 0.001, Fig. 5). *Amblyseius swirskii* significantly reduced thrips number on plants (z = −3.91, p < 0.001; Fig. 5) compared to control, but not *N. cucumeris* (z = −0.85, p = 0.395).

## Discussion

Our results demonstrate that *A. swirskii* and *N. cucumeris* both have the capacity to disseminate *B. bassiana* conidia on plants when foraging. However, *A. swirskii* is more efficient than *N. cucumeris* as they delivered a higher proportion of conidia to thrips colonies.

**Figure 6:**
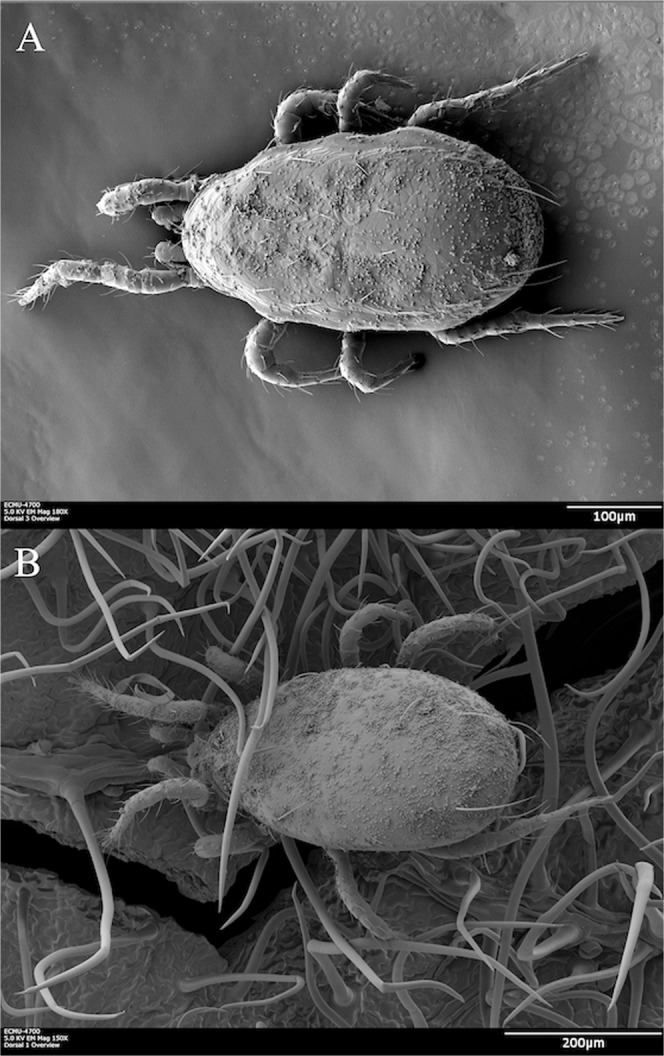
(**A**) *Neoseiulus cucumeris* bearing *Beauveria bassiana* conidia. (**B**) *Amblyseius swirskii* bearing *Beauveria bassiana* conidia, released on a bean leaf. The hair-like structures are dense bean trichomes. We observed and took photos of the specimens using a low temperature scan electron microscope (LT-SEM) with the same method described in Bolton, *et al*.^61^.

There are mainly two ways in which conidia can be dislodged from the predatory mite body and be dispersed on the plant. They can either be actively groomed off by mites or rubbed off on the plant surface when predatory mites move along (Lin *et al*., unpublished data). Grooming, the use of legs to clean the body, has been observed in phytoseiid mites when they encounter potentially pathogenic fungi^24,25^. However, grooming is not efficient to remove all conidia from a mite, especially those located on the dorsal sections of their body. We further showed that *A. swirskii* and *N. cucumeris* mostly dislodged conidia from their body by walking on the plant surface. Indeed, the duration of walking is correlated to conidia removal for both species (Lin *et al*., unpublished data). Trichomes and other structures associated with the surface of bean leaves are likely to facilitate the dislodgement of conidia when mites are walking (Fig. 6B). Foraging predatory mites thus actively disperse *B. bassiana* conidia in the environment.

When mediated by predatory mites, transfer of conidia to thrips can either be a passive or an active process. Conidia are unloaded on plant surfaces and can subsequently passively attach to thrips cuticle when they forage on a contaminated substrate. Alternatively, conidia can be directly transferred from predatory mites to thrips during an unsuccessful predation event involving a physical contact between the two protagonists. Thrips are defensive prey that display counterattack behaviours. They can swing their abdomen to ‘slap’ predatory mites^26^ or secrete irritating anal fluid, which causes predatory mites to withdraw^27^. Moreover, the presence of predatory mites in the vicinity of thrips colonies can affect their behaviour^28^. Following detection of predators, thrips may switch state from stationary feeding to escaping, thereby increasing the probability of coming into contact with spores disseminated on plant surfaces^14^.

The observed differences between *A. swirskii* and *N. cucumeris* in their capacity to disseminate *B. bassiana* conidia to thrips colonies might arise from differences in predator foraging patterns. *Amblyseius swirskii* is classified as subtype III-b generalist predatory mite adapted to mostly living on glabrous leaves, whereas *N. cucumeris* is classified as subtype III-e generalist predatory mite from soil/litter habitats^29^. As a consequence, more *A. swirskii* individuals were recovered from the plant than *N. cucumeris* at the end of our experiment. Such a pattern was also observed on cucumber plants infested with thrips^30^. However, habitat preference cannot alone justify differences in spore dispersal patterns between the two predatory mite species since they both deliver similar numbers of conidia to the plant. *Amblyseius swirskii*, a more efficient predator^30,31^, seems to be better adapted to detect thrips colonies and subdue this type of prey than *N. cucumeris*, as shown by *A. swirskii* suppressing thrips more strongly than *N. cucumeris* in our experimental setup. The difference in proportions of conidia delivered to thrips patches by the two predatory mite species attests to the better capacity of *A. swirskii* to exploit thrips on bean plants. In another study system^32^, showed that the predatory mite *Neoseiulus (Amblyseius) barkeri* Hughes (Acarina: Phytoseiidae) did not increase *B. bassiana* transmission to thrips. *Neosiulus barkeri* is a less voracious thrips predator than *N. cucumeris* with a relatively low capture success when attacking first and second instar larvae of *Thrips tabaci* Lindeman (Thysanoptera: Thripidae)^26^. Furthermore, the level of foraging activity of *N. barkeri* is lower than that of *N. cucumeris*^30^. These results suggest that the foraging capacity of a predator and the strength of its interaction with a prey would be essential determinants of its potential efficiency as a dispersal agent of entomopathogens.

The rate at which *B. bassiana* is contacting its host is crucial in the context of biological control, not only because it is directly linked to the infection rate but also because the viability of conidia is very sensitive to environmental conditions such as UV and humidity^33,34^. Typically, entomopathogens are used like pesticides with single or multiple applications of large quantities of pathogens in crops. However, in some instances, aerial applications are not effective to reach target pests. For example, due to its thigmokinetic behaviour, Western flower thrips are often concealed in plant crevices and flower buds^35^. As a result, spraying fungal pathogens has little effect on thrips infection level^36^. In such circumstances, the capacity of predatory mites in delivering pathogens to thrips colonies could increase disease transmission. In our experiment, the thrips mortality after contacted with pathogens was not evaluated. Nevertheless, it was shown that the LD50 is relative low for technical grade powder of *B. bassiana*: approximately 50 conidia per 2^nd^ instar *F. occidentalis* larva and only 5 per adult^37^.

Our findings about the relative potential of *A. swirskii* and *N. cucumeris* in dispersing *B. bassiana* conidia to thrips are consistent with the conclusion drawn by Zhang *et al*.^18^ who studied a similar biological system on the tropical shrub, *Murraya paniculata* (L.) Jack (Rutaceae), infested by the Asian citrus psyllid, *Diaphorina citri*, in the laboratory. Higher mortality in *D. citri* populations was achieved when *B. bassiana* was delivered by *A. swirskii* rather than by *N. cucumeris*, and compared to *B. bassiana* being sprayed evenly onto plants. We can thus conclude that under our experimental conditions, *A. swirskii* is a better biological control agent because it reduced thrips more strongly and transmitted conidia to a larger number of thrips escaping from predation. However, *N. cucumeris* could show good potential both as a predator and an entomopathogen dispersal agent when used in a different crop-pest association. For example, in greenhouses from temperate regions, it has been shown that *N. cucumeris* showed similar performance as *A. swirskii* as a thrips biocontrol agent under simulated winter conditions^38^.

Finally, how can we apply such a system in a biological control program? Growers periodically release predatory mites and spray *B. bassiana* onto crops to control thrips. The strategy we proposed does not require two separate applications, but solely a premix of *B. bassiana* conidia (technical grade powder) into commercially available predatory mite package (if approved by regulatory agencies)^20^. The predatory mites would likely increase disease transmission rate to concealed pests. The overall quality of a predatory mite species as a pathogen dispersal agent would depend on its capacity to be loaded with conidia, its capacity to resist pathogenic infection and, as shown by the present study, its foraging activity. Predatory mites should be closely associated to the target pest and have the ability to search for, locate and engage in interactions with the pest on the plant, so they can disperse spores on the plant like little pebbles strewn about by Tom Thumb^39^.

## Methods

### The study system

The biological system under study consisted of the entomopathogenic fungus *Beauveria bassiana*, two species of predatory mites *Amblyseius swirskii* and *Neoseiulus cucumeris* as potential fungal dispersal agents and the western flower thrips *Frankliniella occidentalis* Pergande (Thysanoptera: Thripidae) as a resource for both the fungus and the predators. These species share similar habitats (i.e. plants supporting thrips populations) and can coexist in commercial greenhouses applying biological control programs. In a previous study, we showed that *B. bassiana* strain ANT-03 is virulent to thrips (all stages, except first instar larva), slightly virulent to *N. cucumeris* and avirulent to *A. swirskii*^20^. This system thus perfectly fits the profile of a suitable pathogen, vector and host association, in which the pathogen is virulent against host and benign towards the vector^40^.

*Beauveria bassiana* is a generalist entomopathogenic fungus that exploits more than 200 species from most insect orders, with some isolates showing a high degree of specificity^41,42^. Conidia are responsible for infection and natural dispersal by air movement because of their small size (1–3 μm)^43^, by contact with infected hosts or via a dispersal agent^1,14,19^. Conidia adhere to the host cuticle, germinate, penetrate in the host by enzymatic and mechanical processes and next reproduce by exploiting host hemolymph and various host tissues^44–46^. Once host nutrients are depleted, the fungus breaches the cuticle from inside out and sporulates in large numbers^45^. Commercial strains of *B. bassiana* are used for the control of arthropod pests in biological control programs. They are typically sprayed over the crops like pesticides and the probability of contact with the host depends on the spatial distribution of the pests^36,42^.

The two phytoseiid species are generalist predators that actively search for prey^29^. Foraging phytoseiid mites typically respond to chemical cues emitted by plants when attacked by herbivores and move towards infested areas^47^. They are both commercialized and successfully released on vegetable and ornamental crops to control insect pests, including thrips^29^. They both mostly attacked first instar thrips larvae because larger prey successfully counterattack predatory mites^27^. Small and large thrips larvae live together in colonies on plant parts and larger larvae can protect their younger siblings from predation^48^.

*Frankliniella occidentalis* is a cosmopolitan and highly polyphagous insect that feeds on almost every plant parts, from leaves to flower and pods^49–51^, it can also vector a number of plant virus^52^. Their eggs are laid in plant tissues and then go through three stages (two larval and one prepupal stages) before pupation^52^. *Frankliniella occidentalis* can hide in concealed parts of plants where pesticides cannot reach them, and they rapidly develop resistance to chemicals^52,53^.

### Arthropod colonies and fungal inoculum

A colony of *N. cucumeris*, provided by Anatis Bioprotection Inc., was maintained on a factitious prey *Aleuroglyphus ovatus* Toupeau (Acari: Acaridae) while *A. swirskii*, purchased from BioBest Canada, was reared on a diet mixture containing *Carpoglyphus lactis* L. (Acari: Carpoglyphidae) and cherry pollen (Firman Pollen Co., Yakima, WA). *Frankliniella occidentalis* was obtained from a lab colony in Anatis Bioprotection Inc. and reared on California red kidney bean plants *Phaseolus vulgaris* L. (Fabaceae), with cherry pollen supplied *ad libitum* on a weekly basis. All colonies were maintained at 25 °C, 60–70% relative humidity and under a 14 L: 10D light cycle.

*Beauveria bassiana* strain ANT-03 has been registered in North America for greenhouse thrips control. We used the technical grade powder produced by Anatis Bioprotection Inc. containing 5 × 10^10^ conidia g^−1^ for all experiments.

### Prey patch establishment on a plant

**Figure 7:**
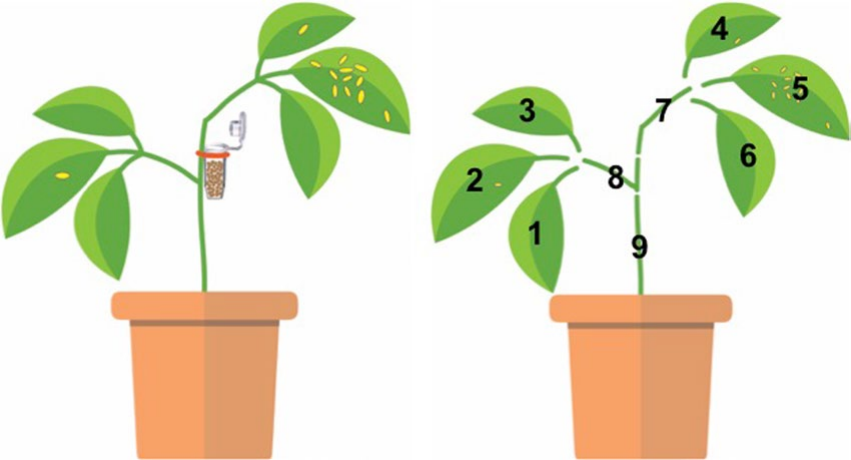
Schematic drawing of the bean plant structure after being trimmed (left). Plant parts (leaflet and stem) are each identified by a number (right). An Eppendorf tube containing predatory mites and fungal conidia to be released was attached in position 8. An example of the spatial distribution of larval thrips is illustrated using yellow oval spots - in this case, thrips are mostly clumped on the oviposition leaflet 5. Drawn by Gongyu Lin with the software Adobe Illustrator.

To test the capacity of predatory mites to deliver *B. bassiana* to thrips, we first established a spatial structure combining plant parts infested or not by thrips. To standardize the structure of a plant, we first trimmed bean plants (approximately 20 cm in height) to two sets of trifoliate (Fig. 7). To create a clumped distribution of thrips larvae on the plant, we enclosed 25 ovipositing female thrips for 24 hours in a clip cage on a single leaf. The clip cage was designed by F. Longpré, London Research and Development Center, Agriculture and AgriFood Canada, and made using a 3D printer. During the oviposition period, female thrips were assumed to have fed and left olfactory cues on the leaf that can further be used by predatory mites to locate the prey patch^54,55^. To avoid potential experimental bias related to leaf age or position, half of the plants had thrips on leaflet 2, the middle leaflet of the old trifoliate, while the second set of plants had thrips on leaflet 5, the middle leaflet of the young trifoliate (Fig. 7). Following oviposition, the clip cage and female thrips were removed from the plant. Four days later, when most eggs had developed into first instar larvae, the suitable prey stage for predatory mites, we released predatory mites loaded with *B. bassiana* on the plant.

### Releasing predatory mites loaded with *B. bassiana* conidia

Adult female predatory mites of various ages were exposed to *B. bassiana* conidia in the commercial rearing substrate (2.5 × 10^9^ conidia g^−1^ substrate) for two hours to obtain maximum conidia load on their body (Fig. 6)^20^. In a modified Eppendorf tube, we put 25 predatory mites with 0.2 g of *B. bassiana* contaminated rearing substrate^20^. The tube was attached on the stem, at equal distance to the base of the petiole of the two trifoliates (Fig. 7). To control for dispersal of *B. bassiana* conidia by air and potential mechanical disturbance during experimental manipulations, a tube containing 0.2 g of *B. bassiana* contaminated rearing substrate was attached to the plant (Control treatment). Each plant was isolated in a paper cylinder and the inner walls and the bottom of the paper cylinder were coated with rings of Tanglefoot^®^ glue to prevent conidia and predatory mites dispersal between experimental units. For each set of plants (leaf 2 *vs*. leaf 5), there were three treatments: control, *B. bassiana* dispersed by *N. cucumeris* and *B. bassiana* dispersed by *A. swirskii*. The experiment was repeated nine times (temporal blocks, n = 9) with two plants per treatment in each block at 25 °C, 60–70% relative humidity and under a 14 L: 10D light cycle. The two blocks where no thrips left on plants were excluded from the analyses of proportion of thrips bearing *B. bassiana* because this parameter cannot be estimated in absence of thrips.

### Recovery of predators and prey

Forty-eight hours after the release of phytoseiid mites, plants were carefully examined to establish the number and spatial distribution of surviving predators and prey. Each of the nine plant parts (Fig. 7) were collected and placed in a 2 oz black solo cup with lid. The cup was filled with carbon dioxide from SodaStream^®^ to stop movement of thrips and predatory mites for the ease of handling and to avoid fungal cross-contamination between individuals. The number of mites and thrips found alive on each plant part was recorded. Thrips mortality was assumed to result from the presence of predators since *B. bassiana* conidia cannot germinate and invade thrips tissues within a 48 h period^20,56^.

### Recovery of *B. bassiana* conidia from prey and plant parts

To detect the presence or absence of *B. bassiana* on living thrips that remained on plants until the end of the experiment, thrips were individually picked with a sterilized toothpick or clean fine brush (sterilized with 75% ethanol and rinsed with 0.1% Tween-80 between samples) and placed in a small Petri dish (Ø 35 mm) containing 2.5 ml of an oatmeal selective media for *B. bassiana*^57^. Petri dishes were examined 10 days later when colony-forming units (CFUs) can be visualized. The proportion of thrips bearing conidia was calculated.

To assess the number of conidia on each plant part following arthropod removal, leaves and stems were cut into small pieces (<2 cm in width or length) and put back into the solo cup. Conidia were washed off by adding 5 ml of 0.1% Tween-80 into each solo cup and the cups were put on a rotary shaker for 2 hours at a speed of 125 rpm^58^. Next, one aliquot of a 0.5 ml suspension was transferred onto the selective media for *B. bassiana*^57^ and CFUs were counted 9 days later. For each plant, we noted the sum of CFUs delivered to the entire plant and, more specifically, the quantity and the proportion of CFUs on the leaf where thrips females laid their eggs.

### Statistical analyses

Our experimental design includes two categorical factors: treatment (3 levels: control, *N. cucumeris* and *A. swirskii*) and leaf where eggs were laid (2 levels: leaf 2 and leaf 5). When either factor was not identified as a significant predictor of the dependent variable following a log-likelihood test, it was eliminated from the initial statistical model to optimize the final model. The number of predatory mites remaining on the plants was analyzed using generalized linear models with negative binomial distribution and with species as a factor. The number of thrips remaining on the plants was analyzed with generalized linear models with negative binomial distribution and with treatment a factor. The proportion of thrips bearing *B. bassiana* was analyzed using generalized linear models with normal distribution with treatment and oviposition leaf as factors. The number of conidia delivered to the entire plant was analyzed with generalized linear models with negative binomial distribution and with treatment and oviposition leaf as factors. The number of conidia on the thrips oviposition leaf was analyzed with both generalized linear models with negative binomial distribution and Kruskal-Wallis tests, depending on whether the residuals were normally distributed or not, determined by Normal QQ-plot^59^. The proportion of conidia on the thrips oviposition leaf was analyzed with generalized linear models. Multiple comparisons were performed with the package ‘multcomp’ with ‘glht’ function and Tukey’s all-pair comparisons method. Kruskal-Wallis multiple comparison tests were performed to compare differences among means when residuals were not normally distributed. All the statistical analyses were carried out with R version 1.0.143^60^.

## Supplementary information

Supplementary Dataset 1

Supplementary Dataset 2

## Data Availability

All data generated or analysed during this study are included in this published article (and its Supplementary Information files).
